# Synthesis Mechanism of an Environment-Friendly Sodium Lignosulfonate/Chitosan Medium-Density Fiberboard Adhesive and Response of Bonding Performance to Synthesis Mechanism

**DOI:** 10.3390/ma13245697

**Published:** 2020-12-14

**Authors:** Xiaodi Ji, Minghui Guo, Li Zhu, Wenxin Du, Hongbin Wang

**Affiliations:** 1 College of Forestry, Northwest A&F University, Yangling 712100, China; yanglinwhb@nwsuaf.edu.cn; 2 Key Lab of Bio-Based Material Science and Technology of Ministry of Education, Material Science and Engineering College, Northeast Forestry University, Harbin 150040, China; gmh1964@126.com (M.G.); nefudwx@hotmail.com (W.D.); 3 Network and Education Center, Yangling Vocational and Technical College, Yangling 712100, China; katrina123789@163.com

**Keywords:** MDF adhesive, chitosan, sodium lignosulfonate, synthesis mechanism, bonding performance, chemical structure, thermal stability, crystalline structure

## Abstract

Environment-friendly medium-density fiberboards (MDFs) prepared using sodium lignosulfonate/chitosan adhesives (L/C) show potential in environment-friendly wood-based panel application. However, the synthesis mechanism of this adhesive and the relationships between synthesis mechanism and bonding performance were not discussed in depth. Herein, the synthesis mechanism of L/C was explored in detail based on characterizations of L/C with different mass ratios of sodium lignosulfonate to chitosan by Fourier-transform infrared spectroscopy, thermogravimetric analysis, and X-ray diffraction. For L/C with different mass ratios of sodium lignosulfonate to chitosan, the corresponding bonding performance was also determined based on characterizations of mechanical and dimensional performance of MDFs. Results showed a 3D network structure of L/C formed through the hydrogen linkages among hydroxyl groups in sodium lignosulfonate and hydroxyl and amino groups in chitosan, amide linkages resulted from reaction between carbonyl groups in sodium lignosulfonate and amino groups in chitosan, and sulfonamide linkages originated from reaction between sulfonic groups in sodium lignosulfonate and amino groups in chitosan. The mechanical performance of MDF was closely related to the 3D network and amino groups of L/C, while the dimensional performance of MDF was negatively affected by sodium lignosulfonate. The MDFs with 1:3 and 1:2 mass ratios of sodium lignosulfonate to chitosan showed superior mechanical properties and comparable dimensional performance with a commercial panel.

## 1. Introduction

Traditional medium-density fiberboards (MDFs) on the market are prepared mainly using formaldehyde-containing adhesives, which causes harm to humans and the environment and further hinders the high-end applications of MDF [1]. Furthermore, these formaldehyde-containing adhesives are made synthetically from non-renewable and non-replenishable petrochemical resources. With the depletion of petrochemical resources, the deterioration of raw materials for synthetic resin has also been becoming an issue of concern. In contrast to these synthetic adhesives, biopolymer-based MDF adhesives, such as soy protein [2], wheat protein, [3] and starch [4], have gained more attention in recent decades, due to their characteristic eco-friendliness and renewability [5]. However, the high costs of these materials and their relatively complicated manufacturing processes along with their poor bonding strengths and water resistances have limited their industrial applications. Recently, chitosan has been recognized world-wide as a potential biopolymer-based wood adhesive, because of its non-toxicity and renewability, which is of great interest to both industries and consumers [6]. Chitosan is a carbohydrate polymer comprising of β-(1,4)-linked 2-acetamido-2-deoxy-d-glucopyranose and 2-amino-2-deoxy-d-glucopyranose units. It is obtained by deacetylation of chitin, which is present as the main structural element of crustacean shells, insect exoskeletons, fungal cell walls, microfauna, and plankton. It is the second most abundant natural polysaccharide, next to only cellulose [7]. Like other traditional amino resin adhesives (e.g., urea-formaldehyde resins and melamine resins), chitosan has reactive amino side groups and hence, it can provide strong adhesion between suitable adherend-like biomass materials [8]. Moreover, use of chitosan along with other hydrophilic polymers can reduce their water affinity due to the formation of hydrogen bonds between their hydrophilic groups [9]. Owing to its anti-microbial properties, chitosan also acts as an environment-friendly preservative [10]. Therefore, chitosan has great potential as an adhesive with multifunctional properties for MDFs.

However, the high cost of chitosan as a wood adhesive restricts its large-scale applications. Hence, blending of chitosan with other low-cost biopolymers is a promising way to reduce its cost. Lignin is one of the most abundant natural polymers, which fills up the voids present in the wood cell wall and bonds together cellulose, hemicellulose, and abutting wood cells. As with traditional phenolic resins, lignin has a three-dimensional polymeric network and many phenolic hydroxyl groups, showing great potential as a cheap, natural MDF adhesive. Lignosulfonate is a lignin derivative, which results from the incorporation of sulfonate groups (SO_3_^−^) into the benzylic carbon of the phenylpropane units of lignin, during the sulfite pulping process [11]. Currently, most lignosulfonate is burned as fuel, which is not only a waste of resources, but also leads to a series of environmental and health problems. Reutilization of lignosulfonate will reduce the amount of the biowaste and add value to lignosulfonate. There are many reactive groups in lignosulfonate, such as sulfonic groups, methoxy groups, hydroxyl groups, carbonyl groups, and phenolic hydroxyl groups, making it an anionic polyelectrolyte that is extremely soluble in water. These reactive groups in lignosulfonate enable it to undergo a variety of chemical reactions, such as sulfonation, oxidation, phenolation, polycondensation, and graft copolymerization. Lately, lignin has found applications as wood adhesives [12,13,14,15,16]. However, lignosulfonate cannot be directly used as an MDF adhesive because of its poor bonding strength and water resistance [17].

Recently, chitosan–lignin blends have been researched for their role in removal of water pollutants, wound dressing, controlled drug delivery, food packaging, and bonding of wood materials [18,19,20,21,22]. In our earlier work [23], the development of lignosulfonate/chitosan adhesives (L/C) as potential cheap and environment-friendly MDF adhesives was presented. The mass ratio of lignosulfonate to chitosan and adhesive amount were preliminary optimized in that work. However, the potential synthesis mechanism of that adhesive was not deeply investigated. The response of bonding performance to synthesis mechanism were not evenly discussed. Therefore, in this study, the synthesis mechanism of L/C was investigated in detail based on characterizations of chemical structure, thermal stability, and crystalline structure of L/C with different mass ratios of lignosulfonate to chitosan. The bonding performance of L/C with corresponding mass ratios of lignosulfonate to chitosan was also determined based on characterizations of mechanical and dimensional performance of corresponding MDFs to analyze the response of bonding performance to synthesis mechanism.

## 2. Materials and Methods

### 2.1. Materials

Chitosan (CAS No. 9012-76-4) was provided by Sun Chemical Technology Co. (Shanghai, China). Its deacetylation degree was more than 95% and its molecular weight was between 100,000 to 150,000 Da. Glacial acetic acid (CH_3_COOH, CAS No. 64-19-7, AR) was supplied by Harbin Kaimeisi Technology Co. (Harbin, China). Sodium lignosulfonate (CAS No. 8061-51-6) was purchased from Wuhan East China Chemical Co. (Wuhan, China). Its sulfonation degree was 1.42 mmol/g. Water distilled in our lab was used for all the experiments. All chemicals were used as received without further purification. Miscellaneous wood fibers (20–80 mesh) comprising a blend of softwood and hardwood fibers from different species including Larix gmelinii, Pinus koraiensis, and Populus davidiana and containing cellulose (46.70 wt%), hemicellulose (29.17 wt%), and lignin (22.39 wt%) were provided by Greater Khingan Range Hengyou Furniture Co. Ltd. (Great Khingan, Foshan, China). The wood fibers were produced by a fiber hot grinding process and dried in an oven at 80 °C to reduce the moisture content from 18% to about 6%. The control group was a commercial MDF with density of 0.8 g/cm^3^ and thickness of 5 mm purchased from Oasis Forestry Industry Co. (Suzhou, China).

### 2.2. Synthesis of L/C

The L/C was synthesized as follows: firstly, chitosan powder and sodium lignosulfonate powder were mixed evenly after being stirred with a rod for a few minutes. Then, an appropriate amount of distilled water was poured into the beaker. The sodium lignosulfonate powder was dissolved immediately and the chitosan powder was dispersed in the sodium lignosulfonate solution. Next, glacial acetic acid solution was dumped into the beaker containing chitosan and sodium lignosulfonate. Finally, the chitosan was completely dissolved and a uniform brown viscous solution was obtained after rapid stirring for 10 to 20 min. The above operations were all carried out at room temperature. The mass ratios of sodium lignosulfonate to chitosan in L/C were 1:0, 3:1, 2:1, 1:1, 1:2, 1:3, and 0:1, respectively. The mass ratio of glacial acetic acid to chitosan was 2:3 and the mass ratio of chitosan to distilled water was 3%.

### 2.3. MDF Preparation

**Figure 1:**
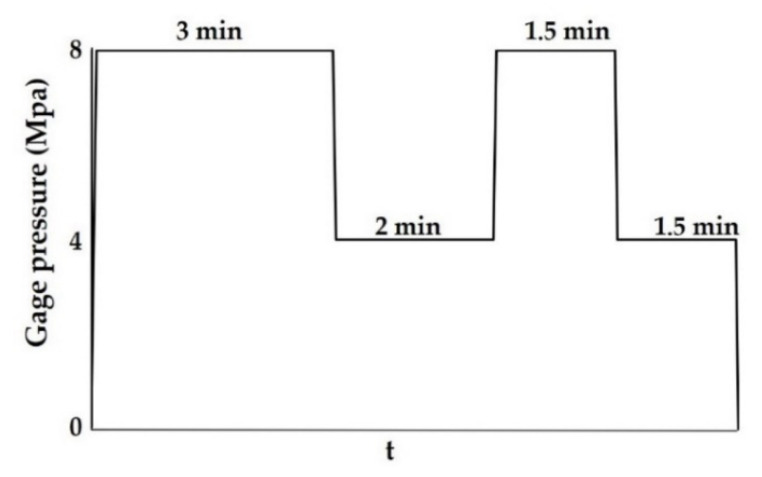
Pre-programmed schedule for hot-pressing process.

The preparation of MDF was as follows: first, the wood fibers were put in a high-speed mixer (SHR-10A, Zhangjiagang Tongsha Plastic Machinery Co., Zhangjiagang, China). Then, the newly-synthesized L/C was poured into the mixture at an adhesive to wood fiber mass ratio of 4% and stirred at 750 r/min for 5 min. Then, the mixture was collected and dried at room temperature until the moisture content dropped to 20%. Thereafter, the mixture was manually spread into a 250 mm × 250 mm forming box and pre-pressed at 1.0 MPa for 1 min to form a slab. Next, the pre-pressed slab was placed between two parallel plane plates (400 mm × 400 mm) of a hot press (Harbin Dongda Wood-based Panel Machinery Manufacturing Co., Harbin, China) and compressed at 170 °C for 480 s employing a pre-programmed process (Figure 1). Finally, the edges were trimmed to obtain the final board size and density of 220 mm × 220 mm × 5 mm and 0.8 ± 0.02 g cm^−3^, respectively. Before the performance test described in the following chapters, MDF was conditioned for 2 days at 40% relative humidity and room temperature.

### 2.4. Synthesis Mechanism Analysis

In order to understand the synthesis mechanism of L/C, the chemical structure, thermal stability, and crystalline structure of L/C were characterized. Fourier-transform infrared (FTIR) spectroscopy (ThermoFisher, Waltham, MA, USA) was used to analyze the functional groups in chitosan, sodium lignosulfonate, and L/C with a Nicolet Magna-IR560 E.S.P. using KBr method, in the spectral range of 650 to 4000 cm^−1^. The spectra were recorded with a resolution of 4 cm^−1^ by accumulating 32 scans. X-ray diffraction (XRD) patterns of chitosan, sodium lignosulfonate, and L/C were collected with a Rigaku D/max 2200 X-ray diffractometer (Rigaku, Tokyo, Japan). The X-ray diffractometer used a Cu Kα radiation source and its scan rate was set to 4° min^−1^. It works at 40 kV and 30 mA, in the 2θ range of 5–40°. The thermal stabilities of chitosan, sodium lignosulfonate, and L/C were measured with a NET-ZSCH-TGA209 thermogravimetric analyzer (NETZSCH, Selb, Germany). The samples (5 mg each in a 70 μL alumina pan) were heated from 40 °C to 800 °C at 10 °C/min and 30 mL/min nitrogen flow rates.

### 2.5. Bonding Performance Test

The bonding performance of L/C could be evaluated from mechanical and dimensional properties of MDFs, which were determined according to the Chinese national standard GB/T 17657-2013. A general mechanical testing machine (CMT5504, Shenzhen Xinsansi Co., Shenzhen, China) was used to evaluate its internal bond strength (IB), modulus of rupture (MOR), and modulus of elasticity (MOE). IB is the ratio of the maximum damage tension perpendicular to the sample surface to the sample area. The low-density surface area of the MDF sample (50 mm × 50 mm) used for IB measurement was polished to improve the surface finish, and then both sides of the sample were glued to steel fixtures with hot melting adhesive. The specimen was pulled apart in the vertical direction using a tensile load to each steel fixture at a crosshead speed of 0.5 mm/min until the specimen failed. MOR is the ratio of the bending moment to the bending modulus under the maximum load, and MOE is the stress–strain ratio generated by the load within the elastic limit of the MDF. MOR and MOE were measured by performing a three-point static bending test on a sample (200 mm × 50 mm) at a speed of 5 mm/min. The dimensional characteristics were determined by the thickness swell (TS) measurement, which is the percentage increase in thickness of the sample (50 mm × 50 mm) after being immersed in water at room temperature for 24 h. The thickness of the sample was measured before and immediately after immersion for 24 h. The MOR and MOE tests were repeated 12 times, and the IB and TS measurements were repeated 8 times to obtain an average value.

## 3. Results and Discussion

### 3.1. FTIR Analysis

**Figure 2:**
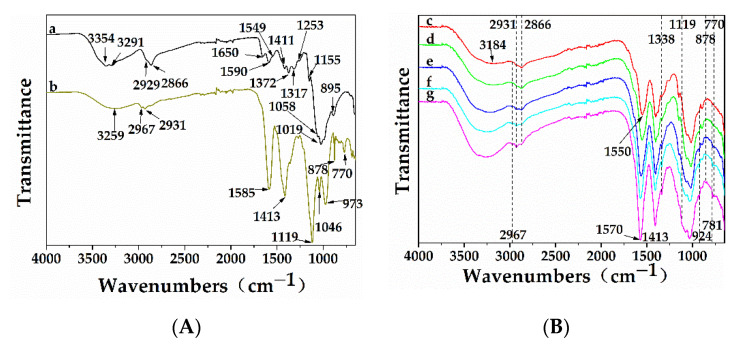
(**A**) FTIR spectra of (a) chitosan and (b) sodium lignosulfonate; (**B**) FTIR spectra of sodium lignosulfonate/chitosan adhesives (L/C) with different mass ratios of sodium lignosulfonate to chitosan: (c) 1:3, (d) 1:2, (e) 1:1, (f) 2:1, and (g) 3:1.

The FTIR spectra of chitosan, sodium lignosulfonate, and L/C are shown in Figure 2. The main peaks in the FTIR spectrum of chitosan were assigned as follows: 3354 cm^−1^ (NH stretching vibrations of the –NH_2_ in primary amines), 3291 cm^−1^ (–NH_2_ stretching vibrations), from 3600 to 3000 cm^−1^ (overlapping of –OH stretching vibrations and –NH_2_ stretching vibrations), 2929 cm^−1^ (–CH_2_ asymmetric stretching vibrations), 2866 cm^−1^ (symmetric stretching vibrations), 1650 cm^−1^ (C=O stretching vibrations of amide I band), 1590 cm^−1^ (NH_2_ in amino group), 1549 cm^−1^ (amide II band, –CN stretching vibrations in acetylated groups), 1411 cm^−1^ (–CH_2_ bending vibrations), 1372 cm^−1^ (–CH_3_ symmetrical deformations in amide groups), 1317 cm^−1^ (CN stretching vibrations of amide III band), 1253 cm^−1^ (amide III band, –NH stretching vibrations), 1155 cm^−1^ (ether bond, C–O–C bending vibrations), 1058 cm^−1^ (C–O stretching vibrations in C–O–C rings), 1019 cm^−1^ (C–O stretching vibrations in C–O–C rings), and 859 cm^−1^ (β-1,4-glycosidic bond) [24,25,26,27,28]. Peaks in the spectrum of sodium lignosulfonate were as follows: 3252 cm^−1^ (–OH stretching vibrations), 2967 cm^−1^ and 2931 cm^−1^ (–CH symmetric stretching vibrations), 1585 cm^−1^ and 1413 cm^−1^ (stretching vibrations of aromatic skeleton), 1119 cm^−1^, 878 cm^−1^, and 770 cm^−1^ (guaiacyl unit, bending vibrations), 1046 cm^−1^ (S=O symmetric stretching of the –SO_3_ groups) and 973 cm^−1^ (out-plane bending vibrations of aromatic skeleton) [28,29,30].

The FTIR spectra of L/C showed some major differences when compared with those of chitosan and sodium lignosulfonate. Two separate peaks at 3354 cm^−1^ and 3291 cm^−1^ and one peak at 3252 cm^−1^ were no longer visible, because a single broad peak appeared in the FTIR spectra of L/C. The broad band was shifted to 3184 cm^−1^ in the FTIR spectrum of L/C with a 1:3 mass ratio of sodium lignosulfonate to chitosan, i.e., red shift occurred. This was attributed to the newly formed intramolecular and intermolecular hydrogen bonds among hydroxyl groups in sodium lignosulfonate and hydroxyl groups and amino groups in chitosan. This band gradually shifted to higher wavenumbers with an increase in the sodium lignosulfonate amount in L/C, due to the fact that the number of hydroxyl groups in sodium lignosulfonate was lower than the number of amino and hydroxyl groups in chitosan, which resulted in lower numbers of hydrogen bonds formed among hydroxyl groups in sodium lignosulfonate and hydroxyl groups and amino groups in chitosan. As the amount of sodium lignosulfonate in L/C increased, the following changes were also observed: an increase in intensities of 2967 cm^−1^ and 2931 cm^−1^ peaks, a decrease in intensity of the peak at 2866 cm^−1^, a gradual increase in the intensity of the peak at 1413 cm^−1^ (stretching vibrations of the aromatic skeleton), a sharp increase in the intensities of the 1119 cm^−1^, 878 cm^−1^, and 770 cm^−1^ peaks (bending vibrations of guaiacyl unit). Moreover, two peaks at 1590 cm^−1^ and 1549 cm^−1^ and one peak at 1585 cm^−1^ were no longer visible, while a sharp peak between 1570 cm^−1^ and 1550 cm^−1^ appeared in L/C. This peak seemed to be a simple overlapping of the peak at 1585 cm^−1^ corresponding to stretching vibrations of the aromatic skeleton, the peak at 1590 cm^−1^ corresponding to NH_2_ in amino groups, and the peak at 1549 cm^−1^ corresponding to CN stretching vibrations in the amide II band. However, in the FTIR spectrum of L/C with a 1:3 mass ratio of sodium lignosulfonate to chitosan, this peak was at 1550 cm^−1^, which was in close proximity to the peak at 1549 cm^−1^ corresponding to CN stretching vibrations in the amide II band, indicating that amide bonds might form as a result of the reaction of carbonyl groups of sodium lignosulfonate and amino groups of chitosan. This peak gradually shifted to higher wavenumbers and got closer to the peak at 1585 cm^−1^ corresponding to stretching vibrations of the aromatic skeleton with an increase in sodium lignosulfonate content in L/C, due to the increasing numbers of aromatics in L/C. A new peak appeared in the spectra of L/C at 924 cm^−1^, which was absent in the spectra of chitosan and sodium lignosulfonate. This peak was assigned to the characteristic amide bond (CO–NH) [31,32]. This confirmed that amide bonds were formed due to the reaction of carbonyl groups of sodium lignosulfonate and amino groups of chitosan. Additionally, two new peaks also appeared in the FTIR spectra of the L/C at 1338 cm^−1^ and 781 cm^−1^, which corresponded to characteristic peaks of sulfonamide groups (S=O and S–N, respectively) [33,34,35]. This suggested that the sulfonic groups in sodium lignosulfonate reacted with the amino groups in chitosan to form sulfonamide groups. The intensities of the peaks at 1338 cm^−1^ (S=O), 781 cm^−1^ (S–N), and 924 cm^−1^ (CO–NH) increased with an increase in the sodium lignosulfonate content of L/C.

Chitosan is a linear polymer, while sodium lignosulfonate is a three-dimensional polymeric network. The chemical connection between chitosan and sodium lignosulfonate is the result of the reaction between carbonyl groups and sulfonamide groups in sodium lignosulfonate and amino groups in chitosan. Hence, it can be speculated that L/C could have a 3D network structure.

### 3.2. Thermal Analysis

**Figure 3:**
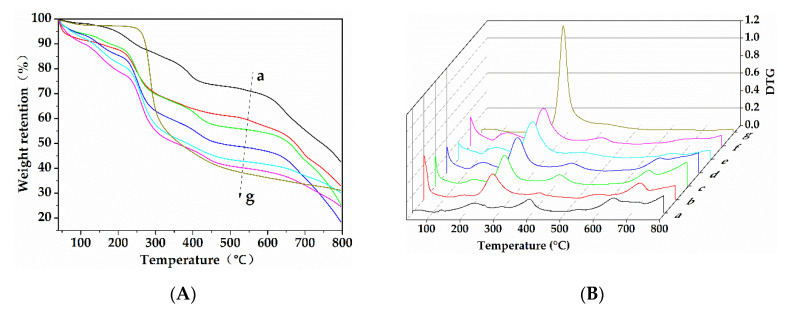
TG (**A**) and DTG (**B**) curves of (a) sodium lignosulfonate, L/C with different mass ratios of sodium lignosulfonate to chitosan: (b) 3:1, (c) 2:1, (d) 1:1, (e) 1:2, (f) 1:3, and (g) chitosan.

**Figure 4:**
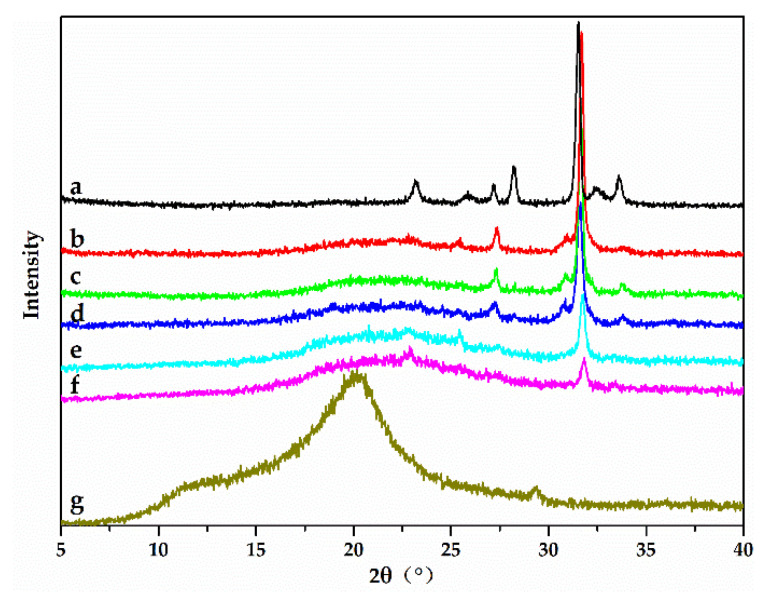
XRD patterns of (a) sodium lignosulfonate, L/C with different mass ratios of sodium lignosulfonate to chitosan: (b) 3:1, (c) 2:1, (d) 1:1, (e) 1:2, (f) 1:3, and (g) chitosan.

The thermogravimetric (TG) and derivative TG (DTG) curves of chitosan, sodium lignosulfonate, and L/C are shown in Figure 3 and the results are presented in Table 1. It can be clearly seen from Figure 4 and Table 1 that the first step of weight loss occurred in the temperature range of 40–100 °C, in which chitosan, sodium lignosulfonate, and L/C lost water molecules.

In the case of sodium lignosulfonate, the weight loss in the temperature range of 100–150 °C was due to the rupture of aliphatic hydroxyl groups, carbonyl groups, and C–C linkages in the side chains of sodium lignosulfonate. Water and carbon dioxide (CO_2_) were formed in this temperature range. The weight loss at 228 °C was ascribed to the decomposition of phenolic compounds (aromatic rings, hydroxyl groups, and alkyl groups). The degradation behavior at 392 °C was ascribed to the thermal degradation of sulfonic groups [36]. The peaks for weight loss above 600 °C were attributed to decomposition, condensation, and secondary thermal cracking of phenolic compounds [37]. As compared to sodium lignosulfonate, the TG-DTG curves of chitosan were simpler. There were only two decomposition stages. The first stage below 100 °C corresponded to loss of water. Maximum weight loss occurred in the second stage, with its peak at 286 °C, which corresponded to the rupture of intramolecular hydrogen bonds and molecular chains of chitosan [38].

The weight loss below 100 °C in L/C corresponded to the loss of water. In the TG-DTG curve of sodium lignosulfonate, the weight loss between 100–150 °C was due to aliphatic hydroxyl groups, carbonyl groups, and C–C linkages in the side chain. Meanwhile, chitosan underwent weight loss slowly and gradually and no distinct weight loss peaks were observed between 100–150 °C. However, the weight loss of L/C increased with a decrease in sodium lignosulfonate content between 100–150 °C. This could be due to the reaction between carbonyl groups of the sodium lignosulfonate side chains and amino groups of chitosan that affected the thermal degradation behavior of L/C within this temperature range. It could also be due the reaction between the thermal degradation products of side chains in sodium lignosulfonate with chitosan that affected the thermal decomposition of chitosan in this temperature range. A weight loss peak at around 240 °C in the TG-DTG curves of L/C could be attributed to the overlapping of the peaks at 228 °C in the TG-DTG curve of sodium lignosulfonate and 286 °C in the TG-DTG curve of chitosan. The weight loss at around 240 °C in L/C increased and the peak was shifted to higher temperatures (280 °C) with a decrease in the sodium lignosulfonate content. It is noteworthy that chitosan lost most of its weight (66.27% of its total weight) at 286 °C. In the case of L/C, although the weight loss in this temperature range increased with increase in chitosan content, the weight loss of L/C with a 1:3 mass ratio of sodium lignosulfonate to chitosan only reached 28.65%, which was significantly less than 66.27%. As mentioned above, the weight loss of chitosan within this temperature range was due to the rupture of intramolecular hydrogen bonds and molecular chains. Hence, the reaction between sodium lignosulfonate and chitosan caused a decrease in the numbers of intramolecular hydrogen bonds of chitosan. This further decreased the weight loss of chitosan within this temperature range. The relative increase in weight loss of L/C within the temperature range of 100–150 °C would also result in a relative decrease of the weight loss of L/C at around 240 °C. The peak at 392 °C in TG-DTG curves of sodium lignosulfonate corresponded to weight loss of sulfonic groups as a result of thermal degradation. Compared to sodium lignosulfonate, this peak in the TG-DTG curves of L/C with a 3:1 mass ratio of sodium lignosulfonate to chitosan shifted to lower temperatures. Moreover, the weight loss corresponding to this peak also decreased. The thermal stability of sulfonamide groups that were newly formed in the reaction between sulfonic groups of sodium lignosulfonate and amino groups of chitosan was lower than those of sulfonic groups. Hence, this peak shifted to lower temperatures. With a decrease in the mass ratio of sodium lignosulfonate to chitosan from 3:1 to 1:3, this peak shifted to higher temperatures, whereas the weight loss continued decreasing. The numbers of sulfonic groups that reacted with amino groups decreased with a decrease in sodium lignosulfonate content. Moreover, the increasing amount of chitosan blocked the further transfer of heat to the sulfonic groups. Therefore, this peak finally shifted to higher temperatures. A weak weight loss peak between 540–570 °C appeared in the TG-DTG curve of L/C with a 3:1 mass ratio of sodium lignosulfonate to chitosan. This peak finally could not be seen with a decrease in the sodium lignosulfonate content of L/C. As for residues beyond 800 °C, most of the L/C blends were less than chitosan and sodium lignosulfonate, meaning that chitosan and sodium lignosulfonate were more thermally stable than L/C at high temperature.

In summary, the TG-DTG curves of L/C were not simply the summation of the individual TG-DTG curves of chitosan and sodium lignosulfonate. The thermal degradation behaviors of L/C differed with differences in mass ratios of sodium lignosulfonate and chitosan in L/C. This was partly due to the reaction between chitosan and sodium lignosulfonate, which affected their respective thermal degradation behaviors.

### 3.3. XRD Analysis

The XRD patterns of chitosan, sodium lignosulfonate, and L/C are presented in Figure 4. Chitosan showed two major peaks at 10.9° and 20.1°, which was consistent with the previous studies [22]. Sodium lignosulfonate showed seven major peaks at 23.1°, 25.9°, 27.2°, 28.2°, 31.5°, 32.5°, and 33.6°. The peak at 31.5° was particularly sharp, which was also consistent with previous studies [39].

In the XRD patterns of L/C, a new weak and broad peak at 22.5° appeared as the chitosan content increased. In the chitosan/sodium lignosulfonate solution, when chitosan was completely dissolved, the crystal structure disintegrated due to dissolution in acetic acid. The crystal structure of chitosan was also affected by the reaction between chitosan and sodium lignosulfonate. Therefore, two peaks at 10.9° and 20.1° disappeared gradually and a new broad peak at 22.5° appeared slowly, which was in agreement with previous studies [40]. Furthermore, the intensities of peaks at 23.1°, 25.9°, 28.2°, 32.5°, and 33.6° gradually weakened. This was due to the overlapping of characteristic peaks of sodium lignosulfonate and chitosan.

The peak at 27.2° in the XRD pattern of sodium lignosulfonate directly disappeared in the XRD pattern of L/C, which was similar to the changes in peaks at 1119 cm^−1^, 878 cm^−1^, and 770 cm^−1^ in the FTIR spectra of L/C. This phenomenon indicated that the disappearance of the peak at 27.2° in the XRD pattern might be related to a potential chemical reaction between guaiacyl units in sodium lignosulfonate and chitosan. A new peak at 30.5° appeared in the XRD patterns of L/C with 3:1, 2:1, and 1:1 mass ratios of sodium lignosulfonate and chitosan and its intensity gradually weakened with increasing chitosan amount, which was similar to the change in the peak at 781 cm^−1^ in the FTIR spectra of L/C. Therefore, there might be a connection between the changes in the peak at 30.5° in the XRD patterns of L/C might and the peak at 781 cm^−1^ in the FTIR spectra of L/C. The change in the peak at 30.5° in the XRD patterns of L/C might further confirm the formation of sulfonamide linkages as a result of the chemical reaction between sulfonic groups of sodium lignosulfonate and amino groups of chitosan, which can be inferred from the change in the peak at 781 cm^−1^ in the FTIR spectra of L/C. The intensity of the peak at 31.5° in XRD pattern of L/C with 3:1 mass ratio of sodium lignosulfonate to chitosan increased compared to that of pure sodium lignosulfonate. The causes of this requires further study.

**Figure 5:**
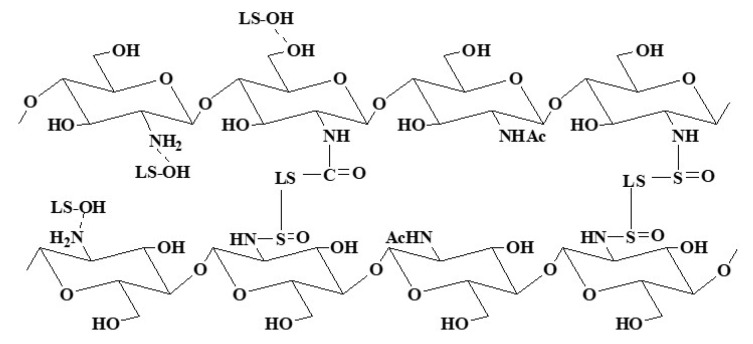
Structure of the L/C (LS refers to sodium lignosulfonate).

From the results of FTIR analysis, TG-DTG analysis, XRD analysis, and previous studies, the chemical structure and synthesis mechanism of L/C could be roughly conjectured (Figure 5). Sodium lignosulfonate is a 3D polymeric network, whereas chitosan is a linear polymer. Sulfonamide and amide linkages were formed by the reaction between sulfonic and carbonyl groups of sodium lignosulfonate with amino groups of chitosan. Hydrogen bonds were also formed between chitosan and sodium lignosulfonate. Therefore, the adhesive transformed into a 3D network structure from the original linear structure of chitosan through these linkages and bonds. The guaiacyl units in sodium lignosulfonate also took part in the reaction.

### 3.4. Bonding Performance Analysis

It can also be concluded from the results of FTIR analysis, TG-DTG analysis, and XRD analysis that the complexity of the 3D network and the numbers of remaining amino groups varied with different sodium lignosulfonate content in L/C, which would result in different bonding properties of L/C. In order to further investigate the response of bonding performance to numbers of amino groups, sulfonamide and amide linkages in L/C, the mechanical and dimensional properties of MDFs using L/C with different mass ratios of sodium lignosulfonate to chitosan as adhesives were determined.

**Figure 6:**
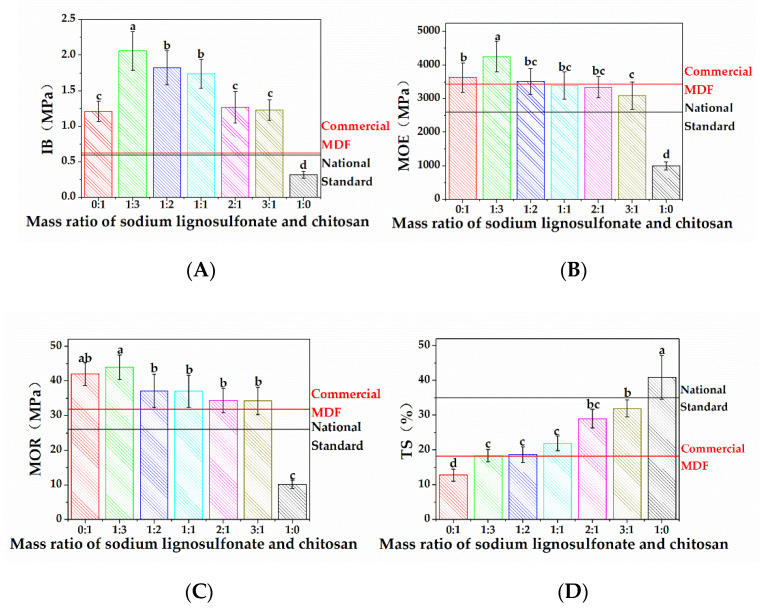
(**A**) Influence of sodium lignosulfonate content on internal bond strength (IB); (**B**) Influence of sodium lignosulfonate content on modulus of elasticity (MOE); (**C**) Influence of sodium lignosulfonate content on modulus of rupture (MOR); (**D**) Influence of sodium lignosulfonate content on thickness swell (TS).

The mechanical and dimensional properties of MDFs L/C with different mass ratios of sodium lignosulfonate to chitosan as adhesives are shown in Figure 6. When the mass ratio of sodium lignosulfonate and chitosan was 1:0 or 0:1—in other words, when pure sodium lignosulfonate or pure chitosan was used as the adhesive—the IB, MOE, MOR, and TS values of MDF were 0.32 MPa, 996.95 MPa, 10.14 MPa, 40.88%, or 1.21 MPa, 3631.1 MPa, 41.96 MPa, 12.73%, respectively. Therefore, the mechanical and dimensional properties of MDF were better when pure chitosan was used as the adhesive than when pure sodium lignosulfonate was used. This indicated that pure chitosan was more suitable as MDF adhesive than sodium lignosulfonate.

However, when the mass ratio of sodium lignosulfonate to chitosan increased from 0:1 to 1:3, the IB, MOE, and MOR of MDF improved, signifying that the mechanical properties of MDF improved when suitable amounts of sodium lignosulfonate were used to replace a portion of chitosan. It could be inferred according to the deacetylation degree of chitosan, sulfonation degree of lignosulfonate, and results of FTIR analysis, TG-DTG analysis, and XRD analysis that when the mass ratio of sodium lignosulfonate to chitosan was 1:3 in L/C, a 3D polymeric network formed with a lot of remaining amino groups. Our earlier work demonstrated that amino groups in chitosan played an important role in bonding with wood fibers [41]. Sodium lignosulfonate could also weakly adhere to wood fibers. Therefore, the mechanical properties of MDFs using L/C with a 1:3 mass ratio of sodium lignosulfonate to chitosan as the adhesive increased compared to MDFs using pure chitosan as the adhesive. With further addition of sodium lignosulfonate in the adhesive, the mechanical properties of MDF did not continue to improve, but instead declined. This phenomenon indicated that a further increase in the complexity of the 3D network and reduction in amino groups which could adhere to wood fibers strongly would not increase the bonding performance of L/C. However, even when the mass ratio of sodium lignosulfonate to chitosan increased further to 3:1, the mechanical properties of this MDF were similar to that of MDF with pure chitosan as the adhesive, indicating that the 3D polymeric network of L/C also played an important role in the mechanical properties of MDFs. The mechanical properties of MDFs deteriorated drastically when the mass ratio of sodium lignosulfonate to chitosan further increased to 1:0—in other words, the MDF was prepared with pure sodium lignosulfonate as the adhesive. This indicated that sodium lignosulfonate itself was not particularly suitable as MDF adhesive.

Compared to pure chitosan as the adhesive, when the mass ratio of sodium lignosulfonate to chitosan in the adhesive was 1:3, the TS of MDF showed a slight increase, indicating a decline in the dimensional property. The trends in the dimensional property were different to the trends in the mechanical properties. This indicated that the dimensional properties of MDFs did not improve despite reaction between sodium lignosulfonate and chitosan, but decreased it due to the hydrophilic nature of sodium lignosulfonate.

In summary, the mechanical properties of MDFs were closely related to the 3D polymeric network and amino groups of L/C. A proper 3D network and large numbers of amino groups were found to improve the mechanical performance of MDFs. The dimensional properties decreased as a result of the addition of sodium lignosulfonate despite the reaction of sodium lignosulfonate and chitosan. The mechanical properties of MDFs using L/C with 1:3 and 1:2 mass ratios of sodium lignosulfonate to chitosan as adhesives were significantly superior to those of the commercial panel, while their dimensional properties were nearly the same. The mechanical and dimensional properties of MDFs using L/C as adhesives were complied with the Chinese national standards for MDF panels (CNS, GB/T 11718-2009, MDF-GP REG).

## 4. Conclusions

The synthesis mechanism and the response of bonding performance to synthesis mechanism of L/C were investigated based on analysis of the chemical structure, thermal stability, and crystalline structure of L/C and mechanical and dimensional properties of MDFs. The results revealed that a 3D polymeric network formed in L/C because of hydrogen linkages among hydroxyl groups in sodium lignosulfonate and hydroxyl and amino groups in chitosan, amide linkages resulted from reaction between carbonyl groups in sodium lignosulfonate and amino groups in chitosan, and sulfonamide linkages originated from reaction between sulfonic groups in sodium lignosulfonate and amino groups in chitosan. The mechanical performance of MDFs was closely related to the 3D network and remaining amino groups, while the addition of sodium lignosulfonate negatively affected the dimensional property of MDFs. The mechanical properties of MDFs with 1:3 and 1:2 mass ratios of sodium lignosulfonate to chitosan were superior to those of the commercial panel, while their dimensional properties were on the same level. The mechanical and dimensional properties of MDFs using L/C as adhesives all met the requirement of the Chinese national standard for MDFs (CNS, GB/T11718-2009, MDF-GP REG). The synthesis mechanism of L/C and the response of bonding performance to the synthesis mechanism could provide a theoretical foundation for further studies that would focus on further cutting the cost of the adhesives, improving the dimensional performance of the MDF, optimizing the hot-pressing process, and exploring the bonding mechanism between the adhesives and MDF.

## Figures and Tables

**Table 1:** Results of TG-DTG curves of (a) sodium lignosulfonate, L/C with different mass ratios of sodium lignosulfonate to chitosan: (b) 3:1, (c) 2:1, (d) 1:1, (e) 1:2, (f) 1:3, and (g) chitosan.

Sample	Stage A		Stage B		Stage C		Residual Amount after 800 °C (%)
	Temperature (°C)	Weight Loss (%)	Temperature (°C)	Weight Loss (%)	Temperature (°C)	Weight Loss (%)	
a	128	0.70	228	8.16	392	16.73	42.62
b	162	2.77	247	21.69	386	11.35	33.22
c	158	4.67	248	23.02	413	10.46	25.67
d	149	8.37	252	26.09	413	10.25	18.33
e	154	10.84	259	27.49	414	9.45	30.55
f	152	12.07	261	28.65	435	9.31	24.73
g	286	66.27	-	-	-	-	31.13
